# Supplementary material for: The Serbian version of the Pandemic-Related Pregnancy Stress Scale (PREPS-SRB)–A validation study
Source: PLoS One. 2025 Feb 4;20(2):e0317909. doi: 10.1371/journal.pone.0317909 (PMC11793785; doi:10.1371/journal.pone.0317909)
Supplement: S1 Appendix — (PDF) [file pone.0317909.s001.pdf]

PREPS – Serbian translation

Пред Вама се налази низ тврдњи. Молимо Вас да сваку тврдњу знаком „✓“ обележите колико се са њом слажете од ,,УОПШТЕ СЕ НЕ СЛАЖЕМ“ до ,,СЛАЖЕМ СЕ У ПОТПУНОСТИ“.

	УОПШТЕ СЕ НЕ СЛАЖЕМ	НЕ СЛАЖЕМ СЕ	НЕМАМ МИШЉЕЊЕ	СЛАЖЕМ СЕ	СЛАЖЕМ СЕ У ПОТПУНОСТИ
Страхујем да због пандемије КОВИД-19 нећу бити довољно припремљена за порођај.	☐	☐	☐	☐	☐
Мислим да ми пандемија КОВИД-19 може угрозити планове везане за порођај.	☐	☐	☐	☐	☐
Прибојавам се да ми због пандемије КОВИД-19 неће бити омогућено присуство партнера на порођају.	☐	☐	☐	☐	☐
Бринем се да ће ме одвојити од бебе после порођаја због епидемије КОВИД-19.	☐	☐	☐	☐	☐
Бринем се да ми због пандемије КОВИД-19 неће бити пружена адекватна контрола трудноће.	☐	☐	☐	☐	☐
Прибојавам се да ми због пандемије КОВИД-19 неће бити пружена адекватна помоћ и нега после порођаја .	☐	☐	☐	☐	☐
Бринем се да због пандемије КОВИД-19 нећу моћи да уносим довољно здраве хране, да нећу имати адекватан сан или да нећу моћи да се бавим физичком активношћу.	☐	☐	☐	☐	☐
Забринута сам да се могу разболети ако одлазим на заказане контроле трудноће код изабраног гинеколога.	☐	☐	☐	☐	☐
Прибојавам се да би КОВИД-19 могао угрозити моју трудноћу (нпр.побачај, претермински порођај,...).	☐	☐	☐	☐	☐
Бринем се да бих ја могла добити КОВИД-19 у породилишту.	☐	☐	☐	☐	☐
Бринем се да би беба могла да добије КОВИД-19 у породилишту.	☐	☐	☐	☐	☐
Страхујем да КОВИД-19 може угрозити здравље моје бебе.	☐	☐	☐	☐	☐
Трудноћа ми даје снагу.	☐	☐	☐	☐	☐
Пандемија КОВИД-19 ми је показала да треба више да вреднујем трудноћу и родитељство.	☐	☐	☐	☐	☐
Трудноћа ми помаже да се носим са тешкоћама.	☐	☐	☐	☐	☐
